# Limitations to Thermoregulation and Acclimatization Challenge Human Adaptation to Global Warming

**DOI:** 10.3390/ijerph120708034

**Published:** 2015-07-15

**Authors:** Elizabeth G. Hanna, Peter W. Tait

**Affiliations:** National Centre for Epidemiology and Population Health, Research School of Population Health. Australian National University, Mills St. Acton, ACT 0200, Australia; E-Mail: aspetert@bigpond.com

**Keywords:** thermoregulation, acclimatization, climate change, climate change adaptation, heat policy, health risks, extreme heat, thermal comfort, heat physiology, limits

## Abstract

Human thermoregulation and acclimatization are core components of the human coping mechanism for withstanding variations in environmental heat exposure. Amidst growing recognition that curtailing global warming to less than two degrees is becoming increasing improbable, human survival will require increasing reliance on these mechanisms. The projected several fold increase in extreme heat events suggests we need to recalibrate health protection policies and ratchet up adaptation efforts. Climate researchers, epidemiologists, and policy makers engaged in climate change adaptation and health protection are not commonly drawn from heat physiology backgrounds. Injecting a scholarly consideration of physiological limitations to human heat tolerance into the adaptation and policy literature allows for a broader understanding of heat health risks to support effective human adaptation and adaptation planning. This paper details the physiological and external environmental factors that determine human thermoregulation and acclimatization. We present a model to illustrate the interrelationship between elements that modulate the physiological process of thermoregulation. Limitations inherent in these processes, and the constraints imposed by differing exposure levels, and thermal comfort seeking on achieving acclimatization, are then described. Combined, these limitations will restrict the likely contribution that acclimatization can play in future human adaptation to global warming. We postulate that behavioral and technological adaptations will need to become the dominant means for human individual and societal adaptations as global warming progresses.

## 1. Introduction

Among the numerous threats to humanity arising from a warming world, the potentially devastating impact of heat exposure has attracted considerable attention in health and policy arenas. The warmth of the 1990s was outside the 95 percent confidence interval of temperature uncertainty, defined by historical variation, during even the warmest periods of the last millennium [1]. The following decade was even hotter, and labelled the “Decade of Extremes” by the World Meteorological Organization [2]. The current decade continues this alarming trend with 2014 being the hottest (globally averaged) year on record [3]. Thermoregulation and acclimatization determine human heat tolerance and vulnerability to heat stress, hence these will be key factors in determining the degree to which humans can accommodate global warming. 

In this paper, we first outline the looming challenges that increased heat exposure presents to humanity despite the development of heat response plans. We then describe the relevant physiological and behavioral processes involved in coping with variable ambient temperatures, the operational thermal range of thermoregulation and protection afforded by acclimatization. Following this, we outline the likely population level limits to acclimatization which will place upper boundaries to human heat tolerance in a warming climate. We conclude with an examination of the limited capacity for acclimatization to protect humans from harm as temperatures continue to climb.

## 2. Global Heat Trends: Current and Projected

Throughout the 135-year period of standardized measurements, the average combined global land and ocean surface temperature for 2014 was the highest on record, and marked the 38th consecutive year (since 1977) that the yearly global temperature was above the 1961–1990 mean global temperature [1]. February 1985 was the last month mean temperature fell below the 1961–1990 mean [4]. The relatively modest average global warming to date of 0.9 °C [5] has delivered a relentless march of new extreme heat records, and inflicted catastrophic human tolls on many continents [4]. Of particular relevance to human health and wellbeing is that since 1979, mean surface air temperatures over land have increased at about twice the rate of temperatures over oceans [6]. Record heat events now outnumber record cold events in the UK by a factor of 10 to 1 [7], and the risk of Australia repeating its 2013 heat record is now 23 fold greater than in the later 19th century[8]. Furthermore, this record heat is fully attributed to anthropogenic climate change [9]. 

It is also noteworthy that the unheralded mass heat-related death events have occurred in regions not accustomed to extreme temperatures, such as Europe (70,000 in 2003) and Russia (55,000 in 2010) [10]. It is self-evident that scope exists for populations habituated to cooler climates to acclimatize to warmer temperatures, and indeed to heat extremes that have historically occurred only in hotter parts of the world. However in a warming world, people living in countries that already routinely experience temperatures over 40 °C, or high 30’s with high humidity, are now facing exposures to temperatures that exceed their acclimatization capacity. Initially, it is the heat sensitive who die. The timeline until global warming delivers climatic conditions that will exceed heat tolerance of the population majority is unknown. An examination of past warming trends and global climate models projections provides concerning evidence of a trajectory that suggests this might occur within a few decades. Risk management principles dictate that national governments need to prepare, and escalate adaptation efforts.

Through the rapid expansion of climate science, the Earth’s climate record now spans 20 million years [11] drawing from paleo records, and diverse specialties such as ice core chemistry (800,000 years) [12]. Standardization of temperature measurements by Departments of Meteorology occurred in 1910 with the advent of the Stephenson’s Screen. Ancient data and many climate models deal with average temperatures that provide baseline data. Whilst these are important, often most damaging to human health are the extreme heat events. In order to prepare for a warmer world, we need to look at both, average and extreme past trends and future projections. 

Global greenhouse gas emissions since 2000 have delivered a global average warming trend that closely matches *Representative Concentration Pathway* (RCP) 8.5, the most dangerous of the four Intergovernmental Panel on Climate Change (IPCC) future climate change scenarios [13]. Climate modelling (using CMIP5 ensembles (Phase 5 Coupled Model Inter comparison Project) [14]) exhibit substantial warming over all terrestrial regions by the 2046–2065 period, with the largest average warming projected over the Northern Hemisphere high latitudes of >4 °C above the 1986–2005 baseline, or about 5 °C above pre-industrial temperatures [15]. Mid latitude models project warming over large areas of North America and Eurasia >3°C in 2046–2065, and by 2081–2100, projected warming exceeds 6 °Cover North America and northern Eurasia, and >4 °C over most other land areas. This is not the distant future, as children born in 2015 will only be 31years old by 2046, 40 years in 2055 and 50 years in 2065. Such warming is unprecedented in human history and will require significant adaptive changes to infrastructure, such as urban and housing design and heat tolerance of bitumen and rail tracks (so they do not warp on hot days), and indeed all human systems.

Ensemble simulations using different climate models (the ECHAM5/MPI-OM) suggest instead, that the strongest changes in near-surface climate will occur in the Sahara, Northern Australia, Southern Australia and Amazonia [16]. Whereas significant variation will continue to occur in the regional- and local-scale warming and extremes that have been already observed due to coastal and land-surface factors, e.g., cloud cover, vegetation, irrigation (with climate-land feedbacks), broad scale warming will continue. For example Australian data reveals that the risk of extreme heat is 25 times greater in dry years than in wet years [17]. These regions are already hot; further warming places serious pressures on societies to identify adaptation options so they may continue inhabiting their homelands. Model discrepancies in location of maximal warming ought not to cloud the key threat that significant life-threatening warming lies ahead in cool and hot regions alike. 

The rapid recent rise in heat extremes underscores our central claim that adaptation to protect human health, and indeed planned mitigation strategies, dangerously underestimate the real health threat of warming in the coming decades, and most critically, in the longer term future, when today’s children turn 50. Turning now to extreme heat events, heat records and prolonged heat waves have increased by more than a factor of ten in some continental regions, including parts of Europe, and the worldwide average is fivefold [18]. Under modest emission scenarios, by the 2040s, monthly heat records globally are projected to be more than 12 times as frequent as in a climate with no long-term anthropogenic warming [18]. In this near-term (2040), irrespective of the emission scenario, robust modelling projects a several-fold increase in the frequency of heat extremes, such that the catastrophic 2003 European heat event will become common [19]. Furthermore, by the end of this century, the coldest summer months will be substantially hotter than the hottest experienced in 2015 [20]. 

Extreme heat events that would occur twice a century in the early 2000s are now expected to occur twice a decade, and in a little over a decade, the return time for more extreme events have reduced from thousands of years in the late twentieth century to about a hundred years [19]. These new events are characterized by long duration, large spatial extent and catastrophic impacts. Warming will be heterogeneous, and regionally dependent upon the local climate system drivers. For example, observed and projected amplifications of the mid-latitude atmospheric planetary quasi-stationary waves preferentially increases the probabilities of heat waves in western North America and central Asia [21]. 

Here, strong agreement exists across all models, that all areas on the globe will endure future temperature extremes far more severe than the recent extreme heat events which have already killed thousands, devastated crops, interrupted infrastructure functioning, caused major urban disruption, and delivered massive economic and development burdens. Societies must prepare, appropriately.

The recent ramping up of heat extremes shows no sign of abating, as many countries remain reticent to curb their emissions of the atmospheric drivers of global warming. Instead of striving to avoid catastrophic warming, global CO_2_ emission rates continue to accelerate [22]. This is despite the scientific certainty of a near linear association of cumulative global CO_2_ emissions and global warming [23]. Implications of this are that the time has now passed for low-cost options to restrict warming to 2 °C; deep emission cuts are now required [13], and an escalation of adaptation effort. This sobering evidence underscores our central claim that adaptation to protect human health, and indeed planned mitigation strategies, dangerously underestimate the real health threat of warming in the coming decades, and most critically, in the longer term future. Hence, an understanding of human heat tolerance, the upper limits to acclimatization and how to safely optimize human heat tolerance, are now needed to face the major heat challenges in hot and cool countries alike. 

## 3. Current Societal Response to Heat Events

Much of the health protection policy focus to date has centered on early warning systems, and response systems which include advisories to avoid the heat, drink fluids, and access cooling where possible [24]. These measures offer useful protection. Several jurisdictions at state and national levels have also introduced a more comprehensive suite of adaptation strategies incorporating the principles and core elements of heat-health action plans as delineated by the World Health Organization [25]. These help to facilitate these actions, such as targeted outreach programs, rescheduling of events and provision of cooling centers [24a, 26]. In 2013, Ahmedabad (India) became the first city in South Asia to release a Heat Action Plan. Following the lead from other global regions, the plan was developed after hundredsof people died in the 2010 heatwave, in which temperatures rose to 46.8 °C [27]. The comprehensive plan involved an early warning system, preparedness strategies including training of medical and community workers to better identify and treat heat-related illnesses, building public awareness of health risks, and coordination of inter-agency emergency response effort when heat waves occurred [28]. 

Early (unconfirmed) press reports suggest this plan contributed to lower death rates in Ahmedabad than cities without heat plans during the May 2015 Indian heat wave [29]. To ensure population health protection through more frequent and more intense heat waves, it is important that such assertions are correct. Melbourne (Australia) is another city prone to heat waves. Following 374 excess deaths during an extreme heatwave in 2009 (three consecutive days exceeding a maximum of 43 °C), a state-wide heat wave plan was developed. However despite slightly lower temperatures (four days over 41 °C), a subsequent heat wave in January 2014, resulted in 167 excess deaths and a doubling of the deaths reported to the coroner [30]. Victorian Government reports attributed the lower death rate to their heat plan [30]. Efficacy of strategies aiming to curtail heat-related morbidity is difficult to evaluate accurately, as differences in intensity and duration between individual extreme heat events render direct comparisons of the ensuing health outcomes challenging, as exposures are always different. 

A promising new metric, the Excess Heat Factor (EHF), derived from normalized climatological variables, facilitates comparisons of localized heat wave intensity, notably, those associated with greatest health and social damages [31]. The EHF enabled a recent trend analysis of heat wave severity in Georgia [32]. The 2006 heatwaves in France resulted in about 4400 fewer deaths than the 2003 event [33], yet an analysis of the relative intensity of the 2003 *versus* the 2006 heatwaves in France suggests the latter heatwave was less intense [34]. Without comparing heat wave intensity, several authors postulated the “lack of excess mortality” of the 2006 event could be attributed to increased awareness in the population to the dangers of heat since 2003, the implementation of a warning system and the measures of the heatwave plan [33,35]. Effective heat plans and greater public awareness clearly contribute to greater localized resilience to heat. A more cautious approach however, would temper reports of success with an analysis of the quantified mortality compared to matching heatwave intensity. A thorough analysis is required to evaluate which existing strategies are most effective, and to determine what additional strategies are required. The task ahead is to minimize mortality prevented *through comprehensive plans* when local communities are confronted with *more dire* (rather than less intense) heat events. 

Despite the development of heat plans, persistently high mortality levels remain. Extreme heat exposure clearly remains a significant health threat, even in the current climate. The health adaptation literature and heat policies fall short of addressing the projected twelve-fold increase in intense and long-lasting heat extremes [18]. Ratcheting up effort will be required to avert further episodes of mass death events. Expectations that currently enacted adaptation measures might offer adequate protection for people and their societal functioning capacity are probably optimistic, especially when acclimatization limitations are factored in. A widespread lack of understanding of acclimatization significance, the mechanisms involved and ultimate limitations, could give rise to dangerous policy oversights. This paper addresses that lack of awareness. 

## 4. The Looming Heat Health Challenge

Throughout human evolutionary history, average global temperatures have predominantly been far cooler than today [36]. Inertia toward emissions mitigation creates potential 21st-century global warming that is comparable in magnitude to that of the largest global changes in the past 65 million years. However these changes are occurring orders of magnitude faster [37]. Humans have not previously witnessed temperatures that are likely to occur in the coming decades, and this presents an unprecedented adaptation challenge [38]. The evolutionary process to physiologically accommodate thermal extremes involves timescales longer than the few decades confronting us. The sobering reality is that the current rate of warming exceeds the capacity of long-living life forms, including humans, to physiologically adapt.

Critical questions therefore facing modern humanity are “Can humans acclimatize to a 2, 3 or 4 degree warmer world?” and “What is the thermal maxima within which modern humans can survive and their societies flourish?”

Human tolerance, and indeed *in*tolerance, to heat exposure is not a new concept [39]. Thermotolerance is the ability of the body and its cellular structures to withstand heat stress that exceeds the optimal temperature range of human performance. Within the physiological literature, usage of the term thermotolerance refers to “a cellular adaptation caused by a single severe but nonlethal heat exposure that allows the organism to survive a subsequent and otherwise lethal heat stress” [40]. However, whilst recognizing the physiological process of thermotolerance occurs at the cellular level, in this discussion of human capacity to tolerate increasingly hot climates, we use the terms *thermal* and *heat tolerance* in the broader public health context to also encapsulate the whole person response to the thermal environment, including capacity to work and find thermal comfort. 

To date, research interest has been largely restricted to thermotolerance studies on military personnel, athletes, and a few studies on occupations exposed to extreme heat, such as mining. Medical and nursing curricula have largely overlooked heat exposure as a population health threat, and instead focused on managing hyperthermia in individuals. (*Hyperthermia* is an increase in core temperature as a consequence of heat gain exceeding the capacity of the body to lose heat [41]). Historically the most common cause of hyperthermia seen in hospitals has arisen from infections and drugs. Exertional heat stress is a recent addition [41] to health text books.

Recent decades of unprecedented heat extremes has increased the inter-decadal global heat-related health burden by 2300% [42]. This has led to a mushrooming of global research effort and policy responses associated with extreme heat exposure [24c, 24d, 27, 43], driven by a recognition of the need to understand and reverse morbidity and mortality trends [24e]. Analyses of heat-related morbidity and mortality statistics have demonstrated two key points. Firstly, considerable variation in heat tolerance exists between regionally dispersed human populations [44], and both between and within sub groups within each population. Secondly, strong consistencies exist in the global patterning of disproportionate vulnerability among certain population subgroups [24f, 44d]. 

*Thermal tolerance* (heat tolerance) can be defined as the range of core (body) temperatures within which human life is sustainable. The definition refers more to a concept than a rigid temperature range. Under a warming climate, it would be instructive to define the upper range of heat exposure that humans can tolerate, that is maintain thermal balance without physiological deficits. However this may not be possible, as a small number of reported cases exist where human beings have tolerated extreme heat stresses well above expected limits, albeit for short periods of time [45].

A suite of epidemiological studies in many national populations have now identified temperature threshold limits, above which ambulance callouts, general practitioner and emergency department presentations, and indeed deaths increase [46]. Temperature threshold limits are profoundly location specific [44b], as resident populations acclimatize to their local climate, some better than others, in a distribution curve. When graphed, the relationship between local temperature and mortality universally follows a U-shaped pattern, with both extreme cold and hot weather precipitating escalation in death rates. Yet, the slope of the curves in each “arm”, and the “Goldilocks” zone of preferred temperature range varies between different cities [47]. Considerable spread exists in the cold temperature curves between cities. For example at cool temperatures, death rates in Sweden are significantly lower than rates for London or Athens [46b], and this is attributed to individual acclimatization, cultural practices, and urban and housing design. For all cities however, the slope of mortality curves in the hot temperatures zones heat are steeper and show less variation of tolerance. This indicates the narrow scope for expanding accommodation to heat through similar mechanisms.

Through a unique combination of physiology, advanced intelligence and ingenuity, humans have successfully inhabited the vast range of thermal environments found between polar and equatorial regions of Earth. The mechanisms underpinning this remarkable achievement, and human capacity to upscale those mechanisms, is now of utmost significance as societies look for solutions to ensure human survival and capacity to continue near global coverage of inhabitation amid global warming.

## 5. Human Thermoregulation 

It is timely therefore to introduce concepts of *thermoregulation* and *acclimatization* to the climate change research and adaptive policy realm. This paper presents a model to assist non-physiologists absorb the salient features of the human physiological response to heat. (Detailed reviews of the physiological processes involved in human thermoregulation are published elsewhere [43b], and publications cited in the reference list.) We demonstrate the limitations to human thermoregulation and acclimatization capacity. The implications are sobering, and ought to shatter any misplaced complacency that humans, and human society can acclimatize and adapt to increasingly severe and more frequent heat extremes. 

Humans are endotherms (a term that has preplaced *homeotherms)* [48], meaning our optimal internal operating temperature (approximately 36.8 °C +/− 0.5) is minimally influenced by the ambient environment [49]. This provides optimal functioning of internal biochemical reactions. Thermoregulation can be defined as a person’s ability to maintain (regulate) a constant core temperature (Tc) within this narrow optimal range. As humans evolved in a cooler climate than today and survived ice ages [36], we have excellent heat generation. Early hominins evolved a suite of complex thermoregulation mechanisms to manipulate heat gain and heat loss via multiple physiological and behavioral processes, enabling them to withstand cold ambient air temperatures (Ta). Evolution of sophisticated cooling mechanisms helped maintain core body temperatures during sustained physical activity. Bipedalism, loss of functional hair and improved sweating capacity facilitated sustained running that made hunting possible, as furred animals lacking sweat glands overheated during the chase [50]. These highly effective heat loss mechanisms enhanced endurance running capacity and provided an evolutionary advantage over other mammals in terms of exploitation of scavenging opportunities when food supplies were scarce [51]. 

This exceptional capacity enables humans to survive and flourish throughout the diverse climate regimes found between the polar and equatorial regions of Earth. Importantly, thermoregulation also allows people to transit across diverse thermal environmental ranges temporarily or permanently. 

Endothermic animals regulate body temperature by autonomic and behavioral thermoeffector responses, predominantly moderated by the hypothalamus [52], which acts as a thermostat [49]. Thermoregulation is therefore achieved through the complex interplay between physiological and behavioural factors. When either of these systems are impaired, thermal tolerance is reduce and sensitivity to heat increases. *Physiological thermoregulation* is capable of precise adjustments in heat balance, but is effective only within a relatively narrow range of environmental temperatures. *Behavioral thermoregulation* relies on an adequate perception of the internal thermal environment and taking appropriate action to modify that thermal environment (e.g., finding a warmer or cooler space, lighting fires, wearing or shedding clothes, increasing or decreasing physical activity as required). These adaptive behaviors expand human thermal tolerance, and enable humans to live in more extreme climates, yet behaviors alone do not provide fine control of body heat balance [53]. 

Physiological thermoregulation refers specifically to the intricate bodily responses that titrate fine adjustments to maintain a core temperature about 36.8 °C (+/− 0.5 °C). When exposed to hot external environments, the physiological component of thermoregulation, described below, is a short term response, which, when repeated over time promotes the development of acclimatization, whereby a person becomes physiologically accommodated to their new climatic environment. Once acclimatized, heat tolerance and physical performance in the heat improve significantly, and time to reaching a state of physical exhaustion can double [54]. 

Heat gain occurs when external heat is absorbed through the skin via transfer directly from the sun and from the atmosphere. Heat is also internally generated as a by-product of basal metabolism and physical activity. Internal organs and viscera within the human body core produce about 70% of the metabolic heat, when resting, whereas this swaps during dynamic exercise, when about 90% of the metabolic heat production occurs in the skeletal muscles [55]. At rest, heat production is ~1.5 W.kg^-1^ [56], which is 105 watts for a 70 Kg person. This can increase to 1400 watts or more during intense exercise [53], a twelve fold increase [55]. Human muscles are surprisingly inefficient. During physical activity 70% to 80% of the energy produced by working muscles is heat, not mechanical work. During physical activity, the body would overheat within four or eight minutes without effective thermoregulatory mechanisms initiating heat loss [55,57]. Thermal balance (homeostasis) exists when heat gain balances heat loss, and is achieved through thermoregulation and acclimatization [43b]. When heat gain exceeds heat loss, heat is stored in body fluids, muscles and bones, resulting in an increase in Tc.

The capacity to shed heat from the body is achieved through heat transfer to the environment via radiation, convection, and evaporation of sweat into latent heat. Conduction of heat is a relatively insignificant mechanism in human thermoregulation [58]. Unlike most mammals who do not have sweat glands, human skin has between 2 and 4 million sweat glands, which contribute to the superb capacity for sustained exercise in warm conditions [52]. Sweat is a solution more dilute than plasma, containing electrolytes (mainly sodium, chloride and potassium), together with other compounds in very small amounts, namely: lactate, urea, ammonia, proteins and peptides [59]. Sweat production in well hydrated trained athletes with 2 m^2^ body surface area can reach 3L/hr, which requires accurate hydration monitoring and fluid replacement to avoid dangerous dehydration. In situations of high sweat rates, electrolyte loss can lead to arrhythmias (irregular cardiac rhythms). The stomach can transfer up to 1.8 L/h of water to the intestines, which have capacity to absorb 1.4–2.2 L/h [60]. Thus a fluid deficit can readily arise when sweat loss exceeds physiological capacity to absorb water. 

Dehydration critically increases risk of heat stress. A hydration deficit of only 1% of body weight elevates core temperature during exercise. As the magnitude of water deficit increases, there is a concomitant graded elevation of core temperature when exercising in the heat, which can reach 0.25 °C for every percent body weight lost [61]. Hence the fundamental public health message “A*void Dehydration*” must be broadly promoted, until the message reaches community saturation [62]. 

Evaporation of sweat from the skin provides the primary mechanism for heat loss to the environment, and this is moderated by Ta, relative humidity and air movement. Higher humidity and temperature gradients across the skin, result in more effective the heat exchange. Conversely, low gradients across the skin in hot, and especially hot and humid environments, make heat shedding increasingly difficult, and increases the discomfiture. 

Heat exposure places the body’s thermoregulatory systems under stress. The term *heat stress* is used to define as the total heat load on the body from metabolic heat production plus external environmental factors; and *Heat Strain* refers to the total physiological stresses resulting from heat stress [43b]. Prolonged heat stress can overwhelm the thermoregulatory system and result in a cascading set of symptoms of increasing severity, (Table 1) ranging from relatively minor to a medical emergency. All require treatment to reduce further heat gain, and normalize hydration and electrolyte levels.

**Table 1:** Cascading heat illness categories.

Heat Illness category	Description
Heat cramps	usually arise in the voluntary muscles of the legs, arms, and abdomen from extensive sweating during exercise leading to sodium deficit; begin as subtle twitches or fasciculation’s [63]; unless treated with fluids and electrolytes, can rapidly progress to widespread debilitating painful muscle spasms
Heat edema	swelling in the periphery (hands and legs); arises from leakage of fluids into the tissues, following prolonged dilatation of the small arteries to facilitate heat exchange
Heat syncope	fainting; arises from reduced blood to the brain through the shunting of blood to dilated cutaneous vessels, postural pooling of blood, diminished venous return to the heart, reduction of cardiac output, and cerebral ischemia [64]. The resultant horizontal positioning (post collapse) helps restore brain blood flow
Heat exhaustion	defined as a mild to moderate illness characterized by inability to sustain cardiac output with moderate (*>*38.5 °C) to high (*>*40 °C) body temperature [55]
Heat injury	moderate to severe illness characterized by organ (e.g., liver and kidney) and tissue (e.g., gut and muscle) injury associated with high body temperature resulting from strenuous exercise and environmental heat exposure, with body temperatures that usually, but not always, are more than 40 °C [55]
Heat stroke	a severe illness characterized by profound central nervous system dysfunction, organ (e.g., liver and kidney), and tissue (e.g., gut and muscle) injury with high body temperatures [55]

Heat stroke is typically divided into exertional and classical (non-exertional) varieties [65]. *Exertional heat stroke* (EHS) predominantly occurs in previously healthy people, exercising in hot and humid climates. EHS is typically seen in small numbers among people playing sport and in occupational settings, notably among those who are not acclimatized. Whereas *classical heat stroke* most commonly affects biologically susceptible individuals, such as infants and young children, the elderly, persons with chronic illness and/or taking medications, as well as persons with alcohol or drug dependencies and with mental illness or neurologic conditions [43b]. The recently observed mass death events caused by extreme heat waves involved both varieties; however the majority of reported deaths occurred due to classical heat stroke. For those who do survive, organ tissue damage can linger post recovery, and many of the long-term consequences of heat stroke are thought to be due to a systemic inflammatory response syndrome [66].

To accommodate potentially lethal heat gain, the ten-fold increase in heat production during physical activity [57] is accompanied by a significant increase in heat loss to the external environment. Heat exchange, during prolonged dynamic exercise under hypothermic conditions, is achieved through dilation of cutaneous (skin) blood vessels which brings hot blood closer to the external environment. Cutaneous blood flow rises contemporaneously with core temperature until the latter reaches about 38 °C. Beyond this physiological limit, core temperatures continues to increase without concomitant increase in skin blood flow, and sweat rates thereafter plateau [67]. The limit of skin blood flow leads to heat gain [54], which is perceived as discomfit, manifests as reduced muscle performance and lethargy [68], motivates heat avoidance behaviors, and results in reduced cardiac function. The sense of fatigue and reduced physical performance is strongly self-protective as it reduces heat generation. 

Studies involving athletes demonstrate that human exercise capacity peaks with (Ta) of 11°C, and declines sharply between 21 °C and 31 °C [69], beyond which the amount of heat generation exceeds the ability to offload heat to the external environment; Tc subsequently rises, and a state of physical exhaustion is reached.

Failure to maintain Tc within a slightly broader range (36.8 °C ± 1.5 °C) is perceived as uncomfortable. Tc diverging beyond this range results in progressive discomfort and deterioration in a range of metabolic and organ functions. Early symptoms of emerging heat stress may include headache, irritability, nausea and vomiting, dizziness, confusion, cramps, shortness of breath, dependent edema (Dependent edema = swelling of feet and ankles, and sometimes hands), and orthostatic hypotension (Orthostatic hypotension = drop in blood pressure when standing, can cause fainting.). When heat gain exceeds heat loss core temperature progressively rise, and leads to cascading symptoms of heat stress and, if not rapidly corrected, to heat stroke at which point reversal becomes progressively more difficult. The result is multi-organ failure, including the brain, and skeletal muscles where, especially in exertional heat stroke, rhabdomyolysis (Rhabdomyolysis = muscle breakdown) and myoglobinuria (Myoglobinuria = a pathological state in which muscle breakdown products appear in the urine which can progress to renal failure) add to the severity of renal dysfunction [70]. This becomes a medical emergency. Chances for survival increase with the rapidity of intervention, which usually requires prompt intensive care management to normalize temperatures and hydration parameters, and to rectify metabolic acidosis. People who reach this state when alone, or in many rural or remote areas, or who are living in disadvantaged regions are unlikely to access the highly technical treatment required to save their life.

Approaching these critical temperatures, a limited set of physiological protective mechanisms come into play. Lethargy, exhaustion and cessation of physical activity is mentioned above. Another key part of this response is the release of *heat shock proteins* which activate to protect some enzyme systems [71] and prevent cellular death. These proteins offer short term protection from cellular damage associated with heat strain such as ischemia, cytokines (short lived proteins released by various cells involved in immune function to act as intercellular messages), and cellular energy depletion [72]. In so doing, they enable excess heat to be stored *temporarily.* Prolonged hyperthermia (one or more hours) however, damages these heat shock proteins, overwhelms their protective response and eventually causes cellular protein damage. Unless the body is cooled, this progresses to the increasingly dangerous situation of degraded organ function, coma and death [73]. The duration of hyperthermia tolerated before tissue damage becomes irrevocable varies according to the intensity of activity generating further heat gain, the underlying health and aerobic fitness status [74], as well as the other environmental factors affecting heating and cooling. This heat shock response is diminished in the aged [75]. 

Due to the very narrow safety margin between mild and dangerous hyperthermia, researchers recommend that physical activity be stopped before subjects reach a critical core temperature of 39.5–40 °C in athletes [76]. Untrained and moderately fit subjects have been shown to reach a state of exhaustion from heat strain during low-intensity exercise with Tc as low as 38 °C [54]. This reinforces the heightened vulnerability to heat stress among the unfit and unwell, and population variance in thermal tolerance and consequent capacity to engage in physical exertion in the heat. 

Beyond the *dangerous* thermal range lies the region where Tc becomes fatal. For the athletic, thermoregulatory decompensation reportedly occurs when Tc reaches 40 to 42 °C [77]. At these temperatures, the thermoregulatory system effectively shuts down. A few documented cases of athletes surviving Tc of 45 °C and higher exist, however duration was very brief. For the non-athletic general population, death is very likely at core temperatures approaching 41 °C [78]. Those with compromised thermoregulatory capacity, for whatever reason, are likely to have less heat tolerance hence a much lower the fatal temperature zone. For all, permanent damage and residual reduction in organ function will begin to occur at lower temperatures. 

For sedated humans, a critical thermal maximum (CTM) has been suggested as a Tc range between 41.6 and 42.1 °C [79]. This high reading reflects an artificial scenario of muscular quiescence, and thus represents a substantively differing situation to daily life, in which routine activities of daily living (ADL) demand muscular heat-generating activity. Thus whilst such research bears limited practical relevance to active humans or functional society, it does however confirm that the upper limitation to human thermal tolerance occurs at a Ta that is increasingly experienced in many parts of the world. 

Whereas some variation has been demonstrated in maximal safety limits of Tc, the primary consideration here is that extreme human tolerances set in artificial situations, such as in conditions of anesthesia, or among athletes and other “extremophiles” who can operate within the upper extreme tail of the heat tolerance distribution curve, is not the core issue. Rather, when considering the future functional capacity of human society in an increasingly warming world, the critical concern is the *population range* of heat tolerance. Human societal functioning requires thermal environments that provide for population performance capacity, and a humane society also demands protection of the physically active and the vulnerable who are at the middle and low ends of the heat tolerance distribution curve. Hence considerations of how we might cope in a world that is 2 or 3 degrees warmer we must take account of more modest thermal ranges of Tc tolerance. 

We found no population level studies on human tolerance of ambient temperatures. However there is a growing literature highlighting reduced worker performance in the heat [80]. To date, our understanding of human heat generation and thermal tolerances is primarily derived from studies measuring the responses of athletes, university students, and military personnel, who are relatively fit and young. Hence caution must be applied when translating findings to the general population, particularly for the aged, cardiac compromised and otherwise unwell, the obese and very young, and people with multiple health risks. 

## 6. Thermoregulation Model

As endothermic animals, the narrow core temperature range maintained by humans is optimal for cellular function. The above discussion outlined the complex physiological processes involved in thermoregulation, the threats arising from excessive heat gain, and the importance of gaining a greater understanding of population-wide heat tolerances. Thermoregulation occurs against multiple pressures arising internally and externally to the human body. These are depicted in the model below, and we expand the discussion in the following sections. The ideal end-point of thermoregulation is thermal homeostasis (steady state) within the optimal range. 

**Figure 1:**
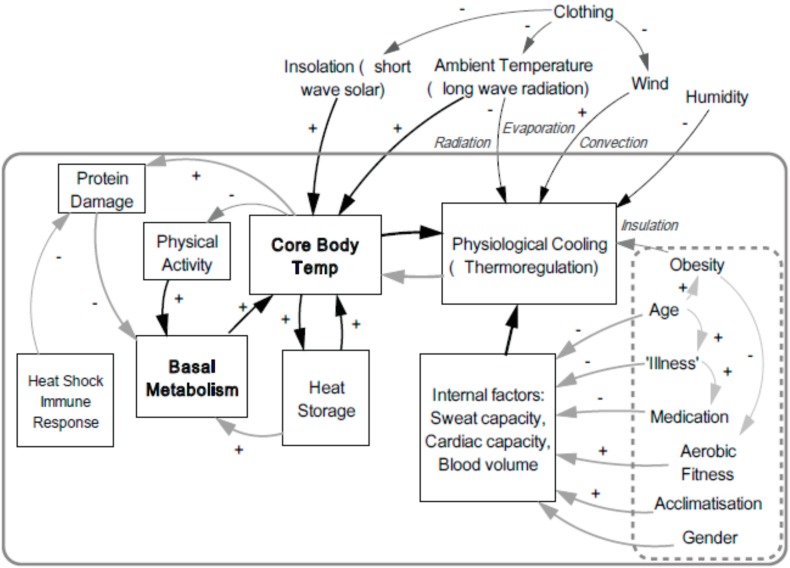

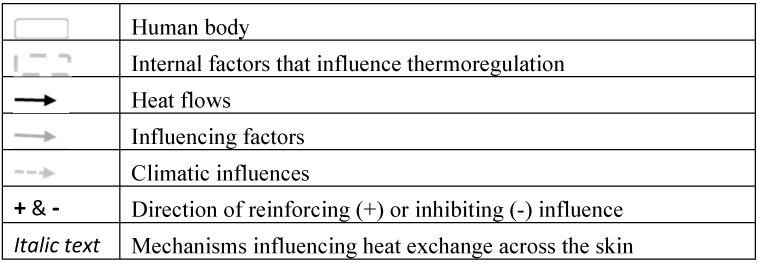
Thermoregulation Model. This model depicts human body heat flows showing the interrelationship of the physiological and external factors that influence thermoregulation.

The Thermoregulation Model (Figure 1) above, sets out a schema for understanding the relationships between a set of internal and external influencing factors on thermoregulation and core body temperature. External factors include climatic variables such as temperature, radiation, wind and humidity. These influences are moderated by clothing and exposed skin surface area. Additional influences on core body temperatures include intensity of physical activity, which raises basal metabolic rate and heat generation, and elevate core temperature. Excess heat is stored in body fluids, muscle and bone. Thermoreceptors detect elevated temperatures and signal responses to initiate cooling, via sweating, and via mechanisms that retard heat generation, such as lethargy which reduces physical activity. Persistently elevated core temperatures lead to protein damage, and the release and activation of protective heat shock proteins, which in turn inhibits basal metabolic rate. Age, illness, and certain medications negatively influence physiological mechanisms involved in thermoregulation, such as sweating, and cardiac capacity. Aerobic fitness level and acclimatization can positively influence, and the influence of gender is still being elucidated. Thus, thermoregulation of core body temperature is subject to complex feedback loops, operating under the influence of many intervening and interconnected mechanisms, all of which are modulated by a set of external and internal factors. Our model presents a simplified version of the major components of these feedback loops. 

In determining human capacity to tolerate heat, the factors depicted in the model above provide the key to more accurately identifying heat vulnerable population groups, and loci for targeting adaptation strategies. Hence these determinants are discussed in greater detail in the following sections.

## 7. Internal Factors

An individual’s physical characteristics, shown in the hatched box (Figure 1), such as obesity, age, illness, medications and fitness [81] strongly influence the efficacy of the key thermoregulation functions of sweating, cardiac system efficiency and blood volume adjustment (discussed below) [82]. Their central role in determining heat vulnerability is demonstrated in studies on multiple continents [83] by their over-representation in heat wave morbidity and mortality statistics. Empirical human experimentation in heat chambers has also reinforced this knowledge, yet gaps persist in the understanding of thermoregulation.

Basal Metabolic Rate (BMR) influences heat generation. It is the base rate of energy used by an organism at rest to function, measured by heat generated per unit time and expressed as either calories/kilojoules (kJ) released per kilogram of body weight, or per square meter of body surface per hour. BMR decreases with age, and is marginally lower (92–97 percent) in women relative to men. For example at age 20, BMR is about 150 and 163 kJ·m^−2^·h^−1^ for women and men respectively. By age 50 this falls to 145 and 150 kJ·m^−2^·h^−1^, and at 80 years, is 130 and 135 kJ·m^−2^·h^−1^ respectively [84].

Age, at either extreme, is associated with lower thermoregulatory and acclimatization capacity. Thermoregulatory efficacy has three age-related phases which approximate those of BMR. Childhood and teens (under 20 s) have immature thermoregulatory capacity, which transitions to full mature capacity in young adults until about 50 years of age, beyond which (over 60 s) thermoregulatory capacity progressively declines [85]. This is principally due to variations in sweating capacity due to sweat (eccrine) gland immaturity in the young, and eventual degraded function in the elderly. The elderly also experience reduced autonomic capacity to titrate cutaneous blood flow, increased prevalence of cardiovascular disease and reduced heat shock protein functionality [86]. Higher baseline and osmotic trigger points for thirst, and blunted thirst and satiety in response to blood volume challenges occurring in older people serves to exaggerate their risks of dehydration, thus further compromising their heat response [87]. Marked in women over 70 [85a, 88], these changes contribute to their high susceptibility to heat.

Heat loss is proportional to the gradient between skin and environment (temperature gradient for dry heat loss and humidity (vapor pressure) gradient for evaporative heat loss), and to the surface area available for heat exchange. Body mass to body surface ratio is therefore important in heat exchange to the environment. Higher surface area to mass ratios are assumed advantageous to better facilitate heat exchange, however the context is relevant. Smaller athletes, with larger surface area to mass ratio, are able to run faster and/or longer in the humid heat than their larger counterparts, while no difference was observed in cool environments [89]. When workload is kept constant in humid conditions, Havenith reported that females, on average had significantly higher body temperatures than males. [90]. In that study, a low body surface to mass ratio advantageously resulted in a lower heat strain. 

Morphology (body shape) and adipose (fat) distribution differs between men and women. Adipose tissue has less heat capacity and transfer of heat across adipose tissue is less efficient (*i.e.*, slower) [91]. By providing a layer of insulation to the body, adipose tissue can, to a certain extent, be protective against environmental heat exchange at both lower and higher external temperatures. However, when Tc is high, the insulating effect of adipose tissue reduces heat loss, and contributes to heat storage and heat stress. This detrimental property elevates heat vulnerability in obese people. In developed countries, obesity is generally positively associated with increasing age, up to the age of 60, thereafter, rates either plateau or decline [92]. Body shape, affected by obesity, also affects cooling potential by altering the surface area to mass ratio [93]. 

Obesity is now regarded as a global epidemic since currently more than 1 billion adults are overweight, and at least 300 million of these are clinically obese. Furthermore, 22 million children under five are estimated to be overweight worldwide [94]. Childhood obesity increases the risk of adult obesity. The combination of increasing prevalence of obesity [95] and a hotter climate, presents a “double whammy” for the individuals involved, as well as for their society. It is possible that these two risk factors are not merely additive in their effect, but rather, multiplicative as increasing heat and increasing obesity prevalence potentiate future population health risks of heat stress. This trend suggests millions more people with diminished functional cooling will be living in a hotter world, thus their capacity for (heat generating) physically activity demanded by engaging in ADLs in hot weather will be limited. 

Several organ systems contribute to thermoregulation, listed in Table 2 with their functions. As with all body systems, these roles and effects are interdependent.

**Table 2:** Thermoregulatory role of key physiological systems.

Physiological System	Role in Thermoregulation
The cardiovascular system	Heart and blood vessels transport heat in blood Differential perfusion shunts blood and heat to skin for cooling Changes in heart rate and blood vessel tone compensate for dilated vasculature and dehydration
Nervous system Higher functions Paleo-brain (hypothalamus) Autonomic (sympathetic/parasympathetic) nerves	Cognitive assessment of risks, planning and taking action Control of thermoregulation Control and modulation of blood vessels, heart, sweat glands
Integumentary system (Skin)	Sweat glands moisten the skin- allowing for evaporative cooling Subcutaneous fat (insulation)
Renal, under influence of endocrine system	Water and electrolyte regulation

Impaired function of the physiological systems listed in Table 2, either individually or combined, can constrain thermoregulation, reduce tolerance to heat extremes and thus, limit acclimatization. Chronic diseases are associated both with aging and with medicine use [96]. Specific categories of medications can interfere with heat tolerance by dampening physiological heat responses, such as vasodilation (dilation of blood vessels) necessary for shunting blood to the skin [97] or by reducing the capacity of the heart rate to increase. Neurological and psychiatric conditions, specifically those that diminish physical and cognitive function, can hamper detection of thermal discomfort, identification and adoption of appropriate behavioral responses. Physical and intellectual disability can similarly result in ineffectual heat responses. 

Heat exposure generates a significant increase in demand for cardiac function. The heart strives to maintain perfusion to vital organs and exercising muscles whilst also perusing significantly dilated vessels in the skin. Blood flow to the skin can reach 7.5 L/min^−1^ (from a resting base of 300 mL·min^−1^) which requires about half the total cardiac output [98]. Meanwhile, exercising muscles also demand significantly increased supply of oxygenated blood. This additional demand creates competition. Furthermore, this increased delivery of blood must occur despite potentially diminished circulating volume through fluid loss (dehydration) as a consequence of increased sweat rates. To maintain blood pressure, the heart must increase its cardiac output, by pumping harder and faster. This increased workload creates strain on the heart. Cardiovascular disease impairs this vital physiological heat response. This additional cardiovascular stress during extreme heat exposure can therefore exacerbate the gap between cardiac capacity to maintain perfusion and extreme cardiac perfusion demands, and precipitate ischaemia (tissues receiving inadequate perfusion), myocardial infarction or cardiac failure, and death. Hence, many heat deaths are recorded as a cardiac related death.

Aerobic fitness improves a person’s ability to manage heat by increasing cardiorespiratory capacity to meet the additional cardiac demands occasioned by internal heat and hot weather [93a]. Subjects with higher aerobic fitness perform longer and tolerate higher levels of hyperthermia (39.2 *vs.* 38.8 °C) than their less fit counterparts in *uncompensable* hot environments [54]. (*Uncompensable* heat is defined as when the evaporative cooling required for the body to achieve a thermal steady state exceeds the maximum evaporative potential of the environment [91]). However this benefit is only observed at temperatures below the body’s maximum operating temperature; it does not extend this upper limit of temperature for safe human functioning. A *persistent* core temperature of 40 °C–41 °C remains lethal for elite athletes.

The physiology literature suggests a lack of consensus on the relative influence of muscle bulk and gender on heat regulation [99]. Muscular bulk gained through regular exercise, can generate more heat than body fat, but requires less exertion to perform certain work. Recent studies identified that changes in core temperature and sweating during exercise in a neutral climate are determined by heat production and body surface area, rather than oxygen consumption, which is often used as a proxy for fitness [100]. This suggests the relationship between fitness and thermoregulatory responses is more nuanced than previously thought, and has the capacity to alter the prevailing wisdom and public health messaging about exercising in the heat. 

Although not present in our model (Figure 1), pregnancy beyond 20 weeks gestation increases BMR, and thus contributes additional physical and metabolic heat load. In pregnant women, heat generation progressively rises until the moment of delivery. The observed variation between individuals is amplified by levels of obesity pre and post-conception [101]. Furthermore, an individual’s bio-susceptibility or indeed resilience, to heat exposure will vary throughout the lifespan [43b]. Vulnerability is greatest among individuals (or subpopulations) with multiple characteristics that are likely to degrade their thermal tolerance and who are highly exposed.

## 8. External Factors

Climate variables influence heat gain and loss across the skin. As a modulator of this effect, clothing creates a microclimate against the skin and thus has a complex effect on heat gain and loss. Clothing can provide insulation from the sun’s radiative heat and from high ambient air temperatures, yet clothing also impedes heat loss to the surrounding environment via radiation, convection and evaporation. Key determinants of this influence are the proportion of skin covered, color (light or dark) and style of the garment (free flowing or in constant contact with the skin), evaporative and breathing characteristics of the fabric involved [102]. Importantly, and as can be readily observed, clothing is one adaptation which is amenable to policy and cultural influence. Thermal properties of clothing, and the relationship between physical performance and clothing is an expanding area of interest, most notably in occupational settings [76]. 

As Ta rises, evaporation of sweat assumes an increasingly important role in thermoregulation among the four mechanisms for heat loss, and becomes the primary mechanism for heat loss [103]. Climatic factors of humidity and wind speed significantly contribute to the temperature and humidity gradients across the skin, and therefore play a major role in determining capacity for effective cooling on hot days. High ambient temperature and high humidity (high water vapor pressure) negatively influence heat loss, whereas higher wind speed has a positive effect on heat exchange with the surrounding airmass [104] through accelerating sweat evaporation.

Evaporation of sweat into the surrounding air removes latent heat from the skin, and thus provides the cooling effect. Sweating saturates the boundary layer of air in contact with the skin. Humid air is already highly saturated, and is therefore less able to absorb extra water from the surface of the skin, making heat shedding to the surrounding air mass less effective. Accordingly hot humid environments are perceived as more oppressive than hot and dry. Wind replaces the boundary layer of air directly in contact with the skin with fresh drier air, which enhances evaporation efficacy and increased heat energy loss to the environment. Hence wind and fans are perceived as refreshing. 

The global trend of increasing heat extremes has already led to the deaths of thousands of people. Since 1971, heat waves have been responsible for most (89.5%) of the total extreme temperature mortality according to the International Disaster Database (EM-DAT) [10a]. Heat exchange slows progressively when Ta exceeds 20 °C, and diminishes significantly when Ta exceed 35 °C. Many parts of the world are experiencing maximum temperatures in excess of 40 °C, and temperatures over 50 °C have been witnessed in parts of Europe, Africa, the Middle East, the Sub-Continent, Australia, the Americas and Asia [105]. More extreme heat records are being set now than in the past, on all continents [2a]. Temperatures of future heat extremes are expected to at least match the regional *average* projected increases [106]. This suggests that by adding an additional 5 °C, Moscow’s future heat extremes would extend the record 40 °C experienced in 2010, to experience 45 °C. Melbourne (Australia) could anticipate 52 °C, whereas heat extremes in India and Pakistan may exceed 55 °C. These temperature extremes will expose millions more people to life-threatening heat stress.

## 9. Thermal Comfort

Heat gain, elevating Tc, arises from basal metabolic rate, physical activity, and exposure to high Ta. Elevations in Tc are detected in specific sites in the body, which send messages to the hypothalamus (thermostat in the brain), which then elicits a series of responses to activate a suite of cooling mechanisms. When hyperthermia is detected, a sense of lethargy acts to inhibit physical activity, thereby reducing additional muscular heat generation. Thermal discomfort prompts behavioral responses such as shedding clothes and seeking shade [107]. 

Thermal comfort plays an important role in thermoregulation. It is described as that condition of mind which expresses satisfaction with the thermal environment [108]. That is, it is an individual’s psychological response to their experience of heat gain as detected at specific thermoreceptors in the skin and core. In addition to the sending messages which elicit the suite of autonomic physiological responses in many organ systems [52] discussed in previous sections, the hypothalamus also communicates with the neocortex [109], wherein messages are received and consciously translated into action. Thus disturbances in thermal comfort motivates behavior to seek immediate relief from excessive heat or cold, and to undertake adaptive responses to establish preferred conditions for heat exchange with the environment. It thus helps to ensure survival [110]. 

Capacity to achieve thermal comfort depends upon several factors: accurate thermal self-assessment, which diminishes with advancing age [111] and cognitive or psychological disability; the degree of autonomy to make choices, such as babies or those who are self- or externally constrained; and the availability of optimal thermal environment choices, such as access to thermal modifiers of shade, water, fans or air-conditioning. Operating in parallel with the autonomic nervous system initiated cooling mechanisms, behavioral responses are therefore also required for effective thermoregulation.

Behavioral elements of seeking thermal comfort are vital for survival, yet in a seemingly perverse irony, can also serve to undermine survival prospects in a hostile thermal environment. We explore this in later sections under our discussions on acclimatization.

## 10. Acclimatization

Acclimatization refers to the physiological response to repeated stimulation to shed heat in high heat conditions. By providing a progressive reduction in physiological strain, thereby improving heat tolerance, it represents a state of improved thermoregulatory capacity, and is thus a process of heat adaptation. Acclimatization confers the important advantage of improving a person’s ability to perform increased work in the heat. (Repeated bouts of exercise in an artificially hot climate, such as a heat chamber, achieves the same the physiological outcome, in which case, the response is called *acclimation* [112].) 

Acclimatization involves a complex multi-organ response delivering a suite of neurological and endocrine responses. Less heat gain occurs as cooling responses activate more quickly and are more effective. Heat transfer to the skin through differential shunting of blood is improved. Increased sweat volume, with concomitant reduction in sweat concentration and time of onset (in relation to increase in body temperature), serve to maintain electrolyte homeostasis (stability). This helps prevent cardiac arrhythmias and other associated health problems. Altered kidney function preserves plasma volume (conserves water) and lessens the debilitating risk of dehydration. These functions combine to perform two key survival techniques: (1) amplification of the cooling response and (2) protection from electrolyte imbalance and dehydration [113]. Cognitively, this enhanced thermoregulatory state can eventually provide thermal comfort when confronted with a different, hotter climate [114]. Hence residents of warm or hot countries can enjoy their local climate.

For people exposed to a hotter climate, acclimatization requires active exposure comprising two to six weeks of daily exercise for about 2hrs (or more) in that novel climate [78b,^115^]. Sessions of *intensified* thermoregulation, for example through exercising in the heat, facilitates more rapid attainment of heat acclimatization, and helps to sustain optimal core temperatures and avoid overheating [56]. Guidelines for athletes recommend 1 or 2 weeks of daily intense exercise [116]. A study involving recreationally active subjects revealed that 75 % of the physiological adaptations were evident within the first 4–6 days of heat-exercise stress exposure [112], yet the time required for these to emerge in more vulnerable population subgroups remains unknown. Opportunity and inclination for the general public to abide recommended protocols involving 100 minutes of sustained exercise, sufficient to increase *T*c to 38.5 °C are likely to be limited [117]. As a deliberative attempt to improve heat tolerance, practices involving exercise in the heat should be approached with caution. The process requires judicious monitoring, as the risk of heat strain is greatest during the early days and weeks of elevated heat exposure challenge. 

Acclimatization complements action taken in the behavioural and technological domains.

### 10.1. Levels of Acclimatization

As the climate continues to warm, it is clear that population level heat adaptation is critical for long term human survival. We have described the importance of acclimatization for an individual’s adaptive response to heat, and two key determinants of acclimatization. Firstly, physiological adaptation occurs in response to repeated heat exposure [56]. Secondly, to achieve high functional heat acclimatization within the minimal period of two weeks, a person needs to actively exercise for several hours per day over two weeks or more to accommodate to their new and hotter climate. Acclimatization, however, is not a binary characteristic, where people are either acclimatized or not. It is graded. Long-term acclimatization provides superior heat adaptation [56]. 

The natural human response to avoid the discomfiture of feeling too hot is to seek thermal comfort, by reducing activity and relocating to cooler surroundings. This lessening of heat exposure extends the time required to attain higher levels of acclimatization. Achieving acclimatization is unlikely to occur without motivation, personal discipline and organizational direction, which may arise through necessity, motivation or inducement. In hot climates, a passive process of partial acclimatization occurs among resident populations via the annual cycling of seasons, which builds naturally through the months leading into the hot season. This applies to the exposed, such as those who undertake regular outdoor activities related to work, sport, shopping or commuting. A direct relationship exists between the level of engagement with, and intensity of physical activity in the heat, and the consequent degree of acclimatization attained. 

At a population level, this results in a continuum of acclimatization. Within this continuum three groupings emerge, each with a differing level of acclimatization. At one end of the spectrum will be the minimally heat exposed and who will achieve minimal acclimatization; partial acclimatization will be achieved by those whose exposure is happenstance; and finally, those who are highly exposed through routine physical activity in the heat are more likely to achieve higher levels of acclimatization. If heat exposure (intensity and duration) and physical exertion and duration are considered as a linear scale, the better acclimatized will reach a point of heat exhaustion and heat stress later, placing them at the upper end of the heat tolerance scale.

However, all three groups remain vulnerable to deleterious health effects of heat exposure. No one is without risk. Their vulnerability differs according to their habituated context, their daily environmental heat exposure, energy expenditure, and their physiological and psychological resilience. Exceeding their customary heat exposure can render even the highly acclimatized in a situation that breaches their thermal tolerance. This is the point at which heat gain exceeds capacity to shed heat, and precipitates dangerous heat storage, and rising Tc. This scenario can arise through intense heat events, bringing higher than usual exposure, through exercise of greater intensity or duration, or a combination of both. Similarly, diminished health such as infections, can also lessen heat tolerance, and result in unanticipated risky heat gain.

The schematic below demonstrates population scaling along the acclimatization continuum, and blurring (as seen in the expanded boxes) between the broad categories of high, partial and low levels.

**Figure 2:**
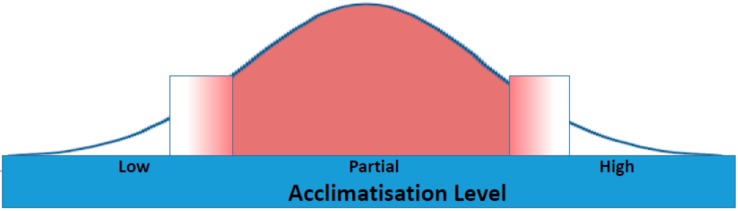
Acclimatization Continuum showing population distribution through three levels, and blurring between groups.

Missing from this schematic is an attempt to insert a temperature scale, as the figure applies to populations living in a range of climates; cool, temperate and hot. Anomalous heat events can occur anywhere on the globe, as revealed by the tragic human toll from the 2010 heatwave in Russia which lasted one and a half months, affected 101 million people and resulted in more than 54,000 excess deaths [118]. The shape of the curve is subject to contextual features of a population. In very cool regions, the population distribution is likely to be skewed towards the lower acclimatization end. Conversely, in hot places such as India, the population average will be skewed towards the higher end. Yet, in all locations, some will tolerate their routinely experienced heat and heat extremes better than others. Their heat tolerance determined according to their personal set of key internal factors described in our Thermoregulation Model (Figure 1). Levels of wealth and personal freedom will further influence relative proportions of populations exposed to uncomfortable and uncompensable heat, and therefore also determine the shape of the acclimatization distribution curve.

In a warming world, regardless of location, the most important intervention one can adopt to reduce physiological strain and optimize performance is to heat acclimatize. To protect health, policies should therefore aim to engineer increasing population acclimatization levels, to effect a shift to the right in the population distribution curve in Figure 2.

### 10.2. Low Acclimatization

Sedentary people, who are usually able to avoid heat exposure, are likely to achieve relatively low levels of acclimatization for their region [56]. By predominantly spending time indoors, people in cool or air-conditioned environments with minimal engagement in exertional outdoor activities, will rarely have their thermoregulatory systems challenged. Their consequent lack of acclimatization will render these people at extreme risk of heat stress during heat waves when they cannot effectively escape the heat, such as during power failures or transport interruptions. Compared to their fellow citizens, their heat vulnerability exists even at relatively low temperatures, and their risk of overheating is high during occasions when they need to venture outside when it is merely hot, not necessarily extremely hot.

### 10.3. Partial Acclimatization

For the population majority, routine daily activities will require physical activity in the ambient heat, yet with some discretionary control over their exposure and their exercise levels. For this group, capacity to cancel or rescheduling exertion for the cooler parts of the day, will minimize high risk heat exposure. Such choices may be individually exercised, or form part of local culture, such as the practice of siestas, or through the regulation of work hours. School children, and those who predominantly work indoors, yet routinely undertake occasional and varying activities in the heat, for example sport, shopping, gardening and commuting are likely to achieve partial acclimatization. As the planet continues to warm and summers become hotter, seasonal partial acclimatization will continue to occur, and population level heat tolerance is likely to incrementally improve, and continue to deliver partial protection, if exposure increase is gradual.

Heat stress risk levels for these groups will depend largely upon discretionary options remaining viable, capacity to rest and minimize physical exertion, wisdom of personal decisions, and access to hydration and cooled environments during extreme heat events, or during episodes of required high exertion during even moderate heat exposure. 

Outdoor workers are likely to attain either partial or high acclimatization, depending on job requirements, discretionary powers and consequent level of heat exposure. In hot countries, people without access to air-conditioning may also be regularly exposed to indoor heat [27], and achieve partial acclimatization towards the upper levels. During extreme heat waves, and without access to cool environments, their exposures may reach hazardous levels, at which time good or partial acclimatization may not offer sufficient protection, as observed during the 2015 Indian heat wave [29]. These examples demonstrate population level acclimatization represents a continuum, rather than discrete categories.

### 10.4. High Acclimatization

At the upper end of the Acclimatization Level spectrum are, for example, outdoor workers, and emergency and essential service (EES) delivery staff whose activities or occupations offer limited discretion about their heat exposure. Workers who secure water supplies or repair power lines during heat extremes, district nurses, firemen, police and ambulance staff, as well as military personnel are included in this group. Whereas some occupations can reschedule work to cooler times, workload for EES personal often intensifies during heat extremes [119]. Repeated heat exposures will repeatedly challenge their thermoregulatory systems, and amplify their capacity for acclimatization. 

Notably, those with poor heat tolerance will elect, if they are able, to avoid occupations involving high heat exposures. Discretionary powers to limit personal heat exposure can also be constrained among those who have responsibility for completing vital tasks (such as fitting a roof to a house), farmers and zoo keepers in charge of animal welfare, and occupations that involve responsibilities to the welfare of others. Through changes in social expectations and negotiation, the option to reschedule some tasks to evening or night time may be achievable. 

In a warming climate, full acclimatization will be a vital component to safe heat adaptation responses, and will determine human capacity to continue functioning, particularly at high physical intensity levels on hot days. Degradation in functional capacity of occupations that offer critical public protection during heat extremes would reverberate throughout society, erode societal capacity to cope with extreme heat events and amplify population vulnerability. An expectation that the EES personnel and others will continue to manage during future heat extremes is a dangerously flawed notion. Heat plans must therefore protect the protectors.

Those whose leisure pursuits entail regular activity in the heat, such as amateur sports people, are also likely to attain high levels of acclimatization, according to the combination of their exercise regularity and intensity, and intensity of heat exposure. However, certain combinations of factors can push these people into the dangerous realm of heat gain. United States’ data reveals that heat related deaths in sports have doubled since the 1970s; many of these were schoolchildren [120].

Another at-risk group are elite sports-people who are pressured by sponsorship obligations to continue playing in extreme heat [121]. Despite their high aerobic capacity, the extreme physical intensity of sports can readily project them into the highest risk categories. The Australian Open Tennis Tournament is played in Melbourne, Australia, in summer. In January 2014, during four consecutive days of temperatures above 41 °C, 970 tennis fans were treated for heat exhaustion [122]. Officials invoked the extreme-heat policy for the first time after the world’s highest ranked tennis players had played for nearly an hour in the sun when the outside temperatures *in the shade* recorded 44 °C [123]. Temperatures inside the stadium were much higher, forcing a reigning champion to describe her game as “like dancing in a frying pan” [124]. Ball-people and players also succumbed to the heat with varying levels of heat symptoms, some severe, and a player hallucinated and collapsed on court from heat exposure. A false sense of confidence in personal heat tolerance can precipitate situations where exposure exceedance relative to their heat loss capacity results in dangerous heat gain, regardless of fitness or acclimatization level. Hence the highly acclimatized also feature in heat related morbidity and mortality statistics [119].

Recently added to the international professional cycling endurance events is the Australian “Tour Down Under”. It is also scheduled in January, which is peak summer in one of Australia’s hottest cities, Adelaide, where temperatures frequently exceed 40°C. Sports promoters who continue to ignore the serious threat posed by heat exposure when scheduling these, and other major international sporting events, in increasingly hot summers reflect highly questionable judgement, and a lack of recognition of the potentially lethal nature of current and future heat extremes. In so doing, they are flouting their legal duty of care [121]. 

## 11. Limits to Acclimatization

Acclimatization offers protection against heat exposure, however that protection is bounded by caveats. As depicted in the Thermoregulatory Model (Figure 1), core temperature is the combined result of BMR, physical activity intensity and duration, and subsequent internal heat generation, the external climatic conditions contributing to heat exchange, level of acclimatization and capacity for dissemination of excess heat to the environment. These all bear on resultant heat gain and determine whether Tc surpasses dangerous thresholds. Thus progression to morbidity and death, or not, depends on the interrelation between heat generation, ambient climatic factors, and the body’s capacity to regulate core body temperature within a safe range. 

Physiological cooling limitations rest with upper limits of key heat loss mechanisms such as maximal sweat rates, and maximal cardiac and perfusions responses. In a warming world, the challenge will increasingly come from capacity to access a thermal environment that does not exceed the person’s capacity to shed excess heat once these physiological limits are reached.

Internal heat generation is a function of physiological mechanisms which abide by specific laws of physics and biochemistry, and will therefore be difficult to modify. Similarly, the immutable, second law of thermodynamics does not allow an object to lose heat to an environment that is hotter. Moreover, vital components within the human physiological systems, for example proteins, are a highly temperature-sensitive [125], and their functionality progressively degrades outside the optimal thermal range. Physiological compensation to continued warming is therefore restricted by biochemical constraints and limits to physiological heat shedding mechanisms. Hence, there is a physiologically constrained absolute limit to the role acclimatization can play in protecting human health. Thousands have succumbed during past episodes of extreme heat, and this will become more dire with the impending increase in heat extremes due climate change [126]. 

There are temperature—humidity combinations beyond which body cooling will not be possible regardless of the state of acclimatization, even with minimal exertion. Hence there exists maximum heat exposure level and duration, beyond which even full acclimatization will not prevent lethal rise in core body temperature. Breaching those upper thermotolerance limits by one of the following: (a) exposure to particularly intense heat; (b) prolonged heat exposure; or (c) exercise of high intensity in the heat, will induce marked hyperthermia.

On hot days, both activity intensity and duration need to reduce in order to prevent Tc rising to unsafe levels. However, performing activities of daily living necessitates (heat generating) physical activity. Often overlooked in heat advisories is the potential for extended heat waves. Discretionary options to cancel or postpone activities erode during protracted extreme heat events; vital societal and domestic activities cannot be stalled indefinitely. Occasions will arise when a person must venture outdoors, regardless of the thermal environment. Aerobic exercise performance degrades in hot environments prior to marked hyperthermia [127], and in certain circumstances, this can result in extending exposures to unsafe heat, particularly among workers and commuters. 

The point of ultimate thermoregulatory limitation will be the temperature and humidity combinations when even rest in the shade will induce dangerous elevations in core temperatures. This combination is likely to be hotter for acclimatized people with more efficient thermoregulatory systems, than for the unacclimatised. But for everyone, there is a set of external climatic conditions beyond which adequate physiological cooling cannot occur. Modelling shows that by 2070, outdoor activity may not be possible in Perth (Australia) for 35–44 days per year compared to 4–6 days in 2010, and that heat-exposed manual labor will be “dangerous” for 15–26 days per year [128]. This pattern will be replicated in all hot regions. Indeed, Abu Dhabi and Dubai are already ‘ahead’ in that they currently experience many days where outdoor work routinely requires policies of self-pacing, and enforced work breaks from 12:30 until 15:00 hours to avoid heat stress among highly acclimatized fit workers [129]. 

An additional limitation to the capacity of acclimatization to protect populations against continued global warming lies in its transitory nature. Once achieved, acclimatization is not a permanent state. Acclimatization capacity decays in the absence of ongoing heat exposure [112]. Furthermore, substantial variation exists between individuals in the time required to acclimatize, and then for acclimatization to decay, and to re-acclimatize. These are influenced by age, health status (especially by physical fitness, obesity (adiposity), or cardiopulmonary diseases), and the type of exposure (*i.e.*, passive heat or heat-exercise exposure) [43b]. Importantly, through transient variations in health status such as tiredness, minor infections or vaccinations, consistency does not exist within an individual. This can be especially problematic for those with variable heat exposure patterns. In situations such as fly-in-fly-out (FIFO) workers, acclimatization decay accrued during absences from the work place re-elevates risk upon return to work if workers attempt to perform at their previous fully acclimatized pace, prior to regaining their pre-departure acclimatization levels. Occupational practices that permit discretionary exertion (self-pacing) and discretionary exposures are health protective, as demonstrated in the Western Australia mining industry [130]. 

In an effort to provide thermal comfort and optimize productive output, employers may instigate behavioral and technological responses to lessen workforce heat exposure, for example, by providing cooled environments. Workplace heat health and safety regimes are to be applauded, however whilst offering protection, an unintended consequence of this practice is the unwitting contribution to delaying or decaying employee acclimatization levels. Indeed some residents in tropical and hot climates report a preference to forgo air-conditioning in order to avoid the awful feeling when they move outside to the heat [131]. Such complexities illustrate the need for deeper understanding of occupational heat exposures, and investigations into effective and enduring work health and safety heat protective policies. 

A proportion of essential outdoor activities may be reduced through greater dependence upon mechanization among societies and industries that can afford such technologies. However mechanization is unlikely to be a feasible solution for billions of people. This option is therefore certain to be limited in scope, and unlikely to occur without detriment to aerobic fitness, which further degrades heat tolerance [69]. 

Indoor and less physically active workers, the unemployed, retired and others such as tourists may lack the ability, opportunity, and even inclination to dedicate sufficient time and effort to achieve full acclimatization. Lack of awareness of the seriousness of risk posed by heat, and therefore understanding of the need to acclimatize is another limiting factor. Achieving thermal comfort for these groups is therefore more likely to occur via technological or behavioral adaptation approaches, such as seeking access to air-conditioning or avoiding external environments during hot times of the day, throughout the hot periods of the year.

## 12. Discussion

The world has thus far experienced warming of less than one degree, yet this relatively small increase raised global mortality of heat deaths by 2300% [132] over one decade. Projected average global warming of another two, three, four or more degrees, and associated longer, more frequent, more extreme and intense heat waves [133] will therefore present major challenges to human capacity to continue to maintain safe core temperatures while undertaking basic activities associated with domestic and professional life [24f]. Human populations will be exposed to a hotter climate; one with heat waves that reach intensity levels never previously experienced. Across many societies, trends in aging populations, increasing sedentariness and obesity [134] are likely to diminish heat tolerance [117], and further potentiate heat exposure risks. 

Most of humanity will become vulnerable to heat stress, some more than others, in the coming decades, and then eventually all. High Ta, high humidity, upper limits to cardiac function required to perfuse vital organs, muscles and skin, and upper levels of sweat rates combine to set boundary limitations on the capacity for heat loss to the environment. Levels of vulnerability will be determined by personal risks associated with direct exposure, a necessity to be physically active, impairment to any of the protective aspects of physiological acclimatization, cognitive or discretionary control to exposure, and access to relief and cooling. Any combination of the multiple features that increase exposure or reduce heat shedding capacity escalates the health risk.

Thus we return to the critical questions facing humanity: “To what extent can acclimatization help individuals survive and continue societal functioning?” Over the next few decades, the key determinants are likely to center on the person’s *capacity to limit exposure* during extreme heat days, the degree of acclimatization *required* to safely undertake necessary activities of daily life within their thermal environment, and individual’s capacity to *achieve* that required level of acclimatization.

Humans have evolved a highly effective capacity to acclimatize to new and changing climates. Complex short-term (thermoregulation) and long-term (acclimatization) physiological responses serve to minimize the influence of climatic variables by providing finely titrated regulation of core temperatures within the physiologically prescribed optimal narrow range. Behavioral and technological adaptations extend the range of thermal tolerance. The combined effect of these has enabled human habitation across a vast range of thermal environments from polar to tropical, including desert regions, and allowed for temporary transition between thermal environments. A direct relationship exists between the extreme nature of the thermal environment, and the necessity of human reliance on behavioral and technological adaptations for survival. Recent hostilities in Yemen starkly demonstrate the precarious nature of human survival when dependent upon other nations for food and water. 

Considerable scope exists among people living in cooler regions of the planet for increased acclimatization to a warmer climate. The same latitude however, does not exist for residents of countries that are already hot, or hot and humid, where heat adaptations and lifestyle modifications already exist, although we can assume that improvements can be made to provide some temporary relief in the near decades. The capacity to significantly upscale acclimatization diminishes among those in hot regions, where acclimatization is already approaching thermal maxima for human tolerance. Survival will require capacity to lessen extreme heat exposures through expansion of existing technical and behavioral adaptations, where possible. These hot nations are at extreme heat risk. That many megacities are situated in hot regions means that billions of people currently have restricted capacity to substantially boost their acclimatization capacity sufficiently to ensure their future health and well-being in a hotter climate. The high humidity endured by many of these megacities will further escalate the risk.

The daily regime involving hours of exercise in the heat, required to achieve full acclimatization, initially incurs significant thermal discomfort and health risks. Seeking to avoid this discomfort by accessing air-conditioning is a natural, and indeed a normalized response in societies where this technology is available and affordable [135]. The resultant delays and decays in acclimatization induced by alternating between heat and air-conditioning therefore presents a major acclimatization challenge for populations in a warmer world [136]. 

Exercising in hot conditions poses one of the greatest challenges to homeostasis of the human body [137]. Physical inactivity is one of the leading risk factors for global disease burden, and accounts for 9% of premature mortality, or >5.3 of the 57 million deaths that occurred worldwide in 2008 [138]. The WHO recommends adults engage in a minimum of 150 minutes per week of moderate intensity activity, or 75 minutes of vigorous-intensity aerobic physical activity to secure cardio- respiratory and musculoskeletal injury protection, and up to 300 minutes per week for added health benefits for those under 65 years [134]. Even to a lesser degree, daily physical activity serves to maintain mobility, independence, and quality of life in older people. Prolonged heat extremes will further reduce physical activity. At a global level, additional heat induced inactivity among this largely sedentary and high-risk population, would have enormous economic and public health implications, especially coinciding with the aging demographics seen in developed nations [117]. Inactivity will impact all ages. Outdoor leisure time involving exercise is temperature dependent, and reduces incrementally beyond the comfort zone [139]. 

Regardless of acclimatization level, physical performance and time to reach physical exhaustion decrease as core temperatures rise [55]. Heat fatigue induced self-pacing reduces productive output in the home as well as in professional spheres [140]. In the forthcoming decades, increasingly severe heatwaves are likely to have detrimental effects on population wellbeing, due to necessary adaptive responses such as limiting physical activity. Prolonged heatwaves will necessitate extended periods of life preserving rest. In such a scenario, personal efficacy and contribution to society will be hampered. Evidence of this is already emerging. For example, thousands of deaths in the prolonged May 2015 Indian heatwave prompted government and union advisories instructing workers to stop work between 11 am and 4 pm [141]. Reductions in collective productive output will inevitably reverberate throughout society, and reinforce the disadvantage of exposed societies and nations.

Indraganti studied behavioural adaptations in Hyderabad, the state capital of Andhra Pradesh (India) where mean maximum outdoor temperatures in summer and the monsoon period are 40.4 °C and 34.2 °C, respectively, in non-heatwave conditions [142]. With high indoor temperatures and inadequate cooling options, only 40% of apartment residents in his study reported being in thermal comfort. Their reported adaptive behaviors included sleeping, “doing something less vigorously” and “sitting in an airy place”. The lives of the poor in many hot countries are therefore already compromised by routine summer heat. Extrapolating this to the global thermal environment projected in future decades gives a scenario in which their situation will worsen, and increasingly, other countries will begin to experience these oppressive conditions. 

The ramifications of population wide reduced physical effort includes significantly reduced productivity across personal, domestic, recreational and economically relevant spheres. For example, occupational field studies with mine works identified that few workers will voluntarily work at a pace that requires sustaining an average heart rate >110 bpm for any length of time [143]. Laboratory studies in a heated chamber confirmed this as a Thermal Work Limit (TWL) such that when subjects were exercised at a level where their heart rates exceeded this threshold, the majority were unable to stabilize heart rate and core temperature [129]. (TWL is defined as the limiting (or maximum) sustainable metabolic rate that euhydrated, acclimatized individuals can maintain in a specific thermal environment within safe limits of both deep body core temperature (<38.2 °C) and sweat rate (<1.2 L/h) [144]). 

Occupational studies demonstrate human performance reductions range between 3% and 50% for temperatures higher than the comfortable level, reaching up to 75 % at Ta of 35 to 37 °C. For office staff, maximal performance is achieved at 23 °C [145] which drops to 70% of the maximum at 30 °C [146], as mental acuity diminishes. For activities involving physical activity, productivity reductions derive also from increasing routine and extreme heat events that elevate heart rate and therefore lower the exercise capacity. Communities experiencing extended periods of broad scale heat induced reluctance (or incapacity) to carry out necessary ADLs of cooking, cleaning, caring, carrying, commuting or working could descend into steep productivity, health and wellbeing decline.

Returning now to consider our groupings along the acclimatization continuum. Under conditions of heat extremes, the ultra-low acclimatized group may continue to function, albeit in vastly constrained lives. For those with minimal daily heat exposure and resultant limited acclimatization, heat avoidance behaviors and technology to provide artificial cooling are likely to constitute their predominant adaptation response and survival strategies. Such sectors of society who spend most of their time in artificially cooled environments are nonetheless required to venture outside in certain circumstances. Their thermoregulatory critical limitation point pivots on the time span required for their transit between cooled environments, and the activity level required to make the transit. If transit is brief, and exposure is time-limited, then higher temperature-humidity combinations may be tolerated in the conduct of their daily routines. 

Importantly, this does not imply a physiological tolerance to those climatic combinations. Untoward events and systems failures, such as the Melbourne 2009 heatwave which caused the power outages to shut down traffic lights, and interrupted rail transport by buckling rail lines [147], are likely to be catastrophic when poorly acclimatized people are unexpectedly detained in extreme heat conditions for an extended length of time. 

For groups with greater exposure, coupled with discretionary powers to modify their personal heat exposure risks, and who are likely to achieve partial acclimatization, behavioral and technological adaptation options will also assume an increasingly vital role in their adaptation response to a hotter climate, as preferences for thermal comfort and safety progressively reduce their exposure. 

An urgent needs exists for improving population wide high level acclimatization. There are very few published studies of long-term acclimatization (or habituation), which occurs over a period of years [43b]. Very high levels of acclimatization will be of critical importance to the adaptive response among people who routinely operate and exercise outdoors. People moving to a climate with more extreme heat, particularly those who are physically active, such as outdoor workers and sportspeople, are at great risk until they acclimatize appropriately for that new climate [148]. Increasingly precarious employment options, and mobile work practices are likely to increase transitory work practices and expand population numbers exposed to thermal shocks. Newcomers may eventually achieve acclimatization, however, during that vulnerable interim, awareness of heat susceptibility and a program of moderated progressive exposure is required to reduce the risk of heat stress. Increasing frequency of occasions where any exercise is unsafe outdoors will inevitably harm societies, and is likely to cause many deaths among those attempting to persevere despite the risks.

Complacency about heat as a serious health risk, and expectations that personal acclimatization could offer adequate protection, has previously been shown to be fatal. This applies to all groups. It is critical therefore that all heat exposed people are informed and recognize that *heat stress risks do not dissipate entirely,* and that when exercising, heat exposure can be lethal even at temperatures below 20 °C [60]. 

Acclimatization to heat is not a static state: and the level of protection it offers is variable, and never complete. When temporary personal factors, such as physical exercise, infections, use of certain medications or drugs and hormonal changes [149] are superimposed on environmental heat exposure, their effects significantly escalate heat sensitivity. Broad education campaigns targeting health practitioners, employers, sporting organizers and community members, about the magnitude of effect of these factors on reducing thermoregulatory capacity is a necessity for populations to be alerted to heightened risk scenarios, and reinforce the need for affected people to adopt additional precautionary measures.

Thus the variable nature of acclimatization further emphasizes the precarious limit that acclimatization can play in preventing heat related morbidity and mortality. All exposed groups will need to apply behavioral adaptation strategies, appropriate to their specific circumstances, in addition to the use of technology. Air conditioning cannot be a global solution, as many activities occur outdoors, and billions of the global population are deprived access [142]. So it is inconceivable to consider that the entire world’s population will be in a position to access air-conditioned environments, all day, throughout all their daily activities. Heat, as a future health burden will inevitably exacerbate existing global inequities within and between countries. Heat exposure and growing inequities are both powerful preconditions for escalations in conflict, at interpersonal and international levels [150]. Departments of Defense recognize the multifactorial threats that climate change present to international security [151].

Global cooperative effort towards stringent mitigation of further greenhouse gas forcing is the only real solution to this dilemma, as the paleo record suggests that with today’s atmospheric CO_2_, the world was about 3 °C to 6 °C warmer [11b]. The alarming lack of ambition in setting enforceable global emission reductions targets therefore necessitates that adaptation must ratchet up. Preparedness for a hotter world is imperative. A concerted multi-agency response, internationally linked, and addressing all sectors of society, with particular focus on the most vulnerable is required as a matter of urgency. 

A clear need exists to boost community resilience to heat exposures. The protective role performed by essential services provides a compelling case for securing their safety as a priority. As warming progresses, every person will need to amplify their acclimatization levels. Well targeted education programs, tailored for policy makers, health care providers, teachers and other groups, and particularly community members, can improve understanding of heat risks and exposure tolerance limits to enable informed responses. Coordinated strategies to actively facilitate the process of enhancing acclimatization across all groups is also required.

This program will need to identify, understand and address barriers, such as:

- Projected increasing intensity of summer heat is likely to lessen willingness and capacity to tolerate exposure to outdoor heat [152].
- Heavy reliance on air-conditioning, and re-scheduling outdoor activities to early and late in the day may reduce heat exposure, and although a sound near term adaptive response, it is unlikely to eliminate heat stress risk, and may amplify risk [139,153].
- Existing heat plans and public health messaging that advise people to always avoid heat exposure [154]. Messaging needs to encourage titrated safe exposures.
- Pressures to maintain high productivity levels, either through self-motivation or externally induced by employers who restrict self-pacing and work-rest ratios may amplify unsafe exposures and risk [80c] [119]. The resultant heat morbidity and mortality may further drive reluctance to acclimatize.
- Indoor and less physically active workers, unemployed, retired and others such as tourists may lack the ability, and opportunity, to dedicate sufficient time and effort to achieve improved acclimatization.
- Lack of awareness of the seriousness of risk posed by heat, and therefore understanding of the need to acclimatize.

In tandem with strategies to improve human resilience to heat exposures, community wide engagement in reducing hazardous exposures through infrastructure, and social changes is needed. Examples include housing design, reconfiguring working hours and altering uniform expectations. The list is exhaustive, and the task ahead enormous. 

Heat protection is extremely complex, and must take into account varying levels of heat sensitivities, acclimatization and exposures to boost resilience and avoid future mass death events. Improved tolerance to heat extremes will be a survival necessity, and a far more nuanced approach is therefore required than existing plans exhibit. 

Research effort needs to shift and expand. The recent focus on quantifying city-specific Ta morbidity relationships has provided valuable information for setting heat warning systems thresholds. However these are too imprecise for the fine grained approaches required. Detailed assessment of the specific characteristics of population heat vulnerability through a better understanding about distinct heat exposure patterning, human physiological and behavioral responses, and variable acclimatization levels among population subgroups is required, beyond the much studied athletic subtype. The very young, the elderly, people with disabilities, people afflicted by obesity or chronic conditions that impact heat tolerance, as well as general population across activity levels, are all at risk of heat stress. As the world rapidly warms, greater research emphasis is needed to identify safe operating levels of core temperatures among these population subgroups. This must be underscored by a deeper understanding of the relationship between climatology of heat intensity and physiological heat response across the population spectrum, coupled with awareness of activity requirements for members of societies to remain physically healthy and productive.

Humanity is on the doorstep of a new future, one with significant and novel challenges, and there is much to learn. A worldwide need exists to identify, trial and test strategies to optimize acclimatization and heat resilience, and to minimize health harm from unaccustomed heat exposure. As a matter of urgency, attention must prioritize strategies to assist vulnerable societies and vulnerable sub-groups to maximize their daily functional capacity, safely.

Countering the global trend towards increasing obesity, and improving population aerobic fitness [155] are two promising strategies. Reversing these strong trends against a backdrop of increasing heat, may present a challenge. But the challenge is not insurmountable.

Increasing population level acclimatization, and physical fitness, will assist heat tolerance, to a point. Climate projections suggest there will be temperature – humidity combinations which will make movement dangerous even for the fully acclimatized. Many parts of the world are already at or beyond, this threshold where sustained physical exercise outdoors is not possible without potentially fatal hyperthermia. If collective mitigation efforts fail to restrict warming to under 2 °C, as is becoming increasingly more likely, all people on Earth will become vulnerable to heat extremes. Even the highly acclimatized will be subject to thermal environments that can overwhelm their thermoregulatory system capacity, when heat gain and heat storage exceed cooling potential. Therefore, temperature induced limitations in capacity for humans to engage in normal daily physical activity, for domestic, commuting, recreational or professional purposes, will become progressively more common, in a warming world. Additionally, the geographical range of this societal limiting feature will progressively expand. Consequently, behavioral and technological adaptations to limit heat exposure will become the dominant means for human individual and societal adaptations as temperatures increase during the remainder of this century. 

The mass death events of the European heatwaves in 2003 and 2010 occurred as the unheralded heat extremes exceeded acclimatization levels and heat protective measures available to the population. Future extreme heat events are now inevitable, and will deliver circumstances of heat exposures never previously experienced. Prevention of mass death events during future warming requires amplification of these protective mechanisms including changes to socio-cultural practices, infrastructure design and technological means. The magnitude of increased mortality, rising 2300% through one decade, testifies the urgency of the problem, and provides critical impetus to mitigate further warming, and ramp up adaptation efforts, without which, the inevitable fate facing humanity is that millions will die from heat exposure. Legislators, policy makers, urban designers, builders, transport engineers, health practitioners, employers, industry, teachers, community members, indeed *everyone* has a role to play. 

## 13. Conclusions

By describing human physiological responses to heat exposure, this paper has provided explanatory power to the observed international consistencies in differential heat vulnerability patterning among population subgroups, most notably, the elderly, those with cardiac impairment and other comorbidities, and the physically active. We outlined the principle human thermoregulatory mechanisms and their role in attaining acclimatization. Our thermoregulatory model depicts the complex interrelationship between ambient climate variables and human core temperatures, and the internal characteristics that moderate heat response and predetermine vulnerability. 

Climate change poses new risks that lie outside our range of experience, and its potential to affect the fundamental social, economic, ecological, and physical systems of communities is already apparent. In this way, climate change and the unheralded heat exposure that accompanies it challenges the concepts, procedures, and scope of conventional approaches to planning, creating a need to rethink and revise current heat policy development, planning methods, and climate adaptation responses among academics, practitioners, industry, policymakers, and throughout civil society as a whole.

Acclimatization to a hotter world is a necessity for survival. However there are limitations. Adaptation to this new world can only partly be achieved through physiological responses. The remainder must derive from orchestrated strategies to reduce exposures via alterations to social, cultural technological and behavioral patterns. Shifts in housing and urban design, clothing, socially accepted behaviors and working hours will be required, as well as reorientations in industry, infrastructure and transport. Such major transformational changes will require decades to achieve, especially in the realm of infrastructure, housing and urban design. The complexity and embedded character of existing systems throughout the socio-political and economic fabric of society, coupled with propensity for inaction suggest that a coordinated multi-frontal approach is essential. This is necessary for human survival and sustained societal functioning in an increasingly hot climate. That process needs to start now. 
